# Bioactivity-Guided Identification of Metabolites from *Syzygium polycephalum* with Antioxidant and α-Glucosidase Inhibitory Activities

**DOI:** 10.3390/molecules31122106

**Published:** 2026-06-15

**Authors:** Ira Rahmiyani, Saeful Amin, Muhamad Insanu, Irda Fidrianny

**Affiliations:** 1 Doctoral Program of Pharmacy, School of Pharmacy, Institut Teknologi Bandung, Bandung 40132, Indonesia; irarahmiyani@universitas-bth.ac.id; 2 Department of Pharmacy, Faculty of Pharmacy, Universitas Bakti Tunas Husada, Tasikmalaya 46115, Indonesia; saefulamin@universitas-bth.ac.id; 3 Department of Pharmaceutical Biology, School of Pharmacy, Institut Teknologi Bandung, Bandung 40132, Indonesia; insanu99@itb.ac.id; 4 Center of Excellence for Innovative Cosmeceuticals and Natural Medicines for Degenerative Diseases-Center for Pharma Valorisation, School of Pharmacy, Institut Teknologi Bandung, Bandung 40132, Indonesia

**Keywords:** antioxidant, alpha-glucosidase inhibition, bioactive metabolites, LC-HRMS/MS, molecular docking, *S. polycephalum*

## Abstract

*Syzygium polycephalum* (Miq.) Merr. & L.M. Perry is an underexplored species within the *Syzygium* genus, traditionally consumed for its edible fruit. However, the potential of its non-edible biomass as a source of bioactive metabolites remains poorly investigated. This study evaluated the antioxidant and α-glucosidase inhibitory activities of different parts of *S. polycephalum* and identified the metabolites associated with these activities using an LC-HRMS-guided approach. The ethanolic leaf extract demonstrated superior phenolic (457.89 ± 12.10 mg GAE/g) and flavonoid (11.08 ± 1.10 mg QE/g) contents with strong antioxidant (DPPH: 683.21 ± 24.54; FRAP: 1338.37 ± 7.04; CUPRAC: 771.91 ± 8.78 mg AEAC/g) and alpha-glucosidase inhibitory activities (52,145.16 ± 801.54 mg AEAGIC/g). LC–HRMS/MS identified four compounds, including chrysin and formononetin. Integrated in silico analyses revealed that chrysin consistently outperformed other metabolites, exhibiting optimal docking scores, favorable absorption, distribution, metabolism, excretion, and toxicity (ADMET) properties, and superior dynamic stability and binding affinity in molecular dynamics simulations. Collectively, these results position chrysin as the dominant bioactive driver and establish *S. polycephalum* leaf as a promising and sustainable source of dual-acting antioxidant and antidiabetic agents.

## 1. Introduction

Diabetes mellitus remains one of the most prevalent metabolic disorders worldwide and continues to pose a major global public health challenge. The pathogenesis of type 2 diabetes mellitus (T2DM) is closely associated with oxidative stress, which contributes to pancreatic β-cell dysfunction, insulin resistance, and the development of chronic complications [1,2]. Excessive production of reactive oxygen species (ROS) disrupts cellular homeostasis and accelerates metabolic deterioration in diabetic patients [3]. Therefore, compounds capable of simultaneously reducing oxidative stress and regulating glucose metabolism is increasingly considered promising candidates for the prevention and management of diabetes.

One widely recognized therapeutic strategy for managing type 2 diabetes is the inhibition of alpha-glucosidase, the intestinal enzyme that breaks down complex carbohydrates into absorbable glucose. This mechanism effectively attenuates postprandial hyperglycemia and enhances glycemic control, as demonstrated in systematic reviews and meta-analyses of alpha-glucosidase inhibitors such as miglitol and acarbose [4]. However, currently available synthetic inhibitors, including acarbose and miglitol, are frequently associated with gastrointestinal side effects such as abdominal discomfort and diarrhoea. These limitations have stimulated growing interest in natural products as safer alternatives. Numerous studies have demonstrated that plant-derived phenolic compounds and flavonoids exhibit significant α-glucosidase inhibitory activity together with strong antioxidant properties, highlighting their potential as multifunctional antidiabetic agents [5,6,7]. 

Within natural antioxidant sources, the genus *Syzygium* (Myrtaceae) is notable for its high polyphenolic content. Several species, namely *S. caryophyllatum*, *S. cumini*, and *S. luzonense*, have shown both antioxidant and antidiabetic potential through in vitro assays, including DPPH and inhibition of α-amylase and alpha-glucosidase [8,9,10]. A recent comprehensive review further highlighted the nutritional and functional potential of *Syzygium* species as sources of health-promoting phytochemicals [11]. Nevertheless, most available studies summarized in these reviews primarily focus on edible fruits and their nutritional value, while many species and plant parts remain poorly investigated.

Limited information is available on *Syzygium polycephalum* (Miq.) Merr. & L.M.Perry, an understudied species endemic to Java and Kalimantan. Traditionally, it is used to treat scabies, acne, dysentery, diabetes, and to delay premature aging [12]. The fruit has been used empirically by communities in Sulawesi to strengthen teeth. However, the fruit represents the only edible part of the plant, and its consumption has declined substantially due to the species’ limited geographic distribution and highly seasonal fruiting pattern (maximum once annually for 3 months). The sustainability challenges of fruit availability make it unsuitable as a consistent pharmaceutical raw material source. Notably, while the cortex has been reported to be rich in tannin-type phenolics [13], the leaf of many *Syzygium* species are known to accumulate flavonoid derivatives that may confer different bioactivity profiles and potentially stronger enzyme inhibitory properties. Plant leaf is recognized as major sites of secondary metabolite biosynthesis and frequently accumulate high levels of phenolic compounds and flavonoids that function as protective agents against environmental stress. These metabolites have been widely associated with antioxidant activity and the inhibition of key enzymes involved in carbohydrate metabolism [14,15].

Despite the growing body of research on the *Syzygium* genus, comprehensive investigations focusing on the phytochemical composition and bioactivity of non-edible parts of *S. polycephalum* remain very limited. In particular, the specific metabolites responsible for the antioxidant and antidiabetic activities of this species have not yet been systematically characterized. Advances in analytical techniques such as liquid chromatography coupled with high-resolution mass spectrometry (LC-HRMS/MS) enable detailed metabolite profiling and facilitate the identification of bioactive compounds within complex plant extracts [16]. Furthermore, computational approaches, including molecular docking, provide valuable insights into the interaction between natural compounds and therapeutic targets such as α-glucosidase [17]. However, metabolite-level identification of bioactive compounds from non-edible biomass of *S. polycephalum* using LC-HRMS-guided analysis remains largely unexplored.

Therefore, the present study aims to identify bioactive metabolites of *S. polycephalum*, integrating in vitro bioactivity assays, LC–HRMS/MS-based metabolite profiling, molecular docking analysis, and in silico absorption, distribution, metabolism, excretion, and toxicity (ADMET) prediction to identify potential bioactive metabolites and elucidate their interactions with relevant biological targets. In particular, molecular docking simulations were employed to further assess the stability and dynamic behaviour of selected protein–ligand complexes, complementing docking results. By revealing the phytochemical composition and biological potential of non-edible plant parts, this research contributes to the discovery of multifunctional natural compounds and supports the sustainable utilization of plant biomass for antidiabetic drug development.

## 2. Results

### 2.1. Plant Part Selection and Preliminary Bioactivity Screening

All plant materials used in this study were authenticated based on their morphological and microscopic features. The identification confirmed the species as [*Syzygium polycephalum* (Miq.) Merr. & L.M. Perry]. Detailed results of the plant identification are provided in the Supplementary Materials (Certificate S1). The initial screening of different parts of *S. polycephalum* (leaf, twig, seed, and fruit) revealed significant variations in phytochemical content and biological activities. The data on total phenolic and flavonoid content, antioxidant (DPPH, FRAP, CUPRAC), and AGI activities among the ethanol extracts of the four plant parts showed significant differences (*p* < 0.05). Generally, in numerous studies, α-glucosidase inhibitory (AGI) activity is reported as either the percentage of inhibition at a specific concentration or the IC_50_ value. In contrast, this study introduces a novel approach by expressing AGI activity as milligrams of acarbose equivalent α-glucosidase inhibitory capacity (AEAGIC) per gram of sample. This approach indicates that the inhibitory capacity of 1 g of the sample is comparable to a specified amount of α-glucosidase inhibition, equivalent to a certain milligram dose of acarbose. Table 1 showed that the ethanolic leaf extract displayed the highest TPC and TFC, correlating strongly with its superior antioxidant performance in CUPRAC, FRAP, and DPPH assays, as well as potent alpha-glucosidase inhibition.

### 2.2. Successive Extraction, Fractionation and Subfractionation of Leaf Extracts

Successive extraction of *S. polycephalum* leaf using solvents of three different polarities (n-hexane, ethyl acetate, and ethanol) revealed distinct phytochemical distributions (Table 2). The ethanol extract exhibited the highest total phenolic content (TPC), significantly surpassing those of n-hexane and ethyl acetate extracts. For total flavonoid content (TFC), the ethyl acetate extract showed the highest values, followed by ethanol and n-hexane extracts. The combined fractions (CF1, CF2, CF3), which were derived from the process of fractionating the ethanol extract, demonstrated varying levels of TPC and TFC. Notably, CF3 exhibited the highest values of both TPC and TFC, indicating that this fraction holds the greatest potential for bioactivity.

Bioactivity assessment (Table 3) revealed that the ethanol extract possessed remarkably superior activities across all assays (DPPH, FRAP, CUPRAC, and AGI). Antioxidant values were approximately 20-fold higher than those of ethyl acetate and n-hexane extracts. VLC of the ethanol extract yielded 21 fractions, which were subsequently grouped into three combined fractions (CF1, CF2, and CF3) based on similar TLC profiles. Among these, CF3 demonstrated the greatest TPC and TFC (Table 2), as well as superior bioactivities (DPPH, FRAP, CUPRAC, and AGI), indicating that CF3 contains a high concentration of bioactive phenolic compounds. CF2 exhibited moderate AGI activity, though with high variability, suggesting that it contains a mixed composition of compounds, some of which have weaker or inconsistent bioactivity. On the other hand, CF1 showed minimal activity across all assays, suggesting the enrichment of less bioactive or non-phenolic compounds in this fraction. The selection of CF3 for further subfractionation was therefore justified by its balanced profile of phenolic richness and potent dual antioxidant–antidiabetic activities, indicating the presence of bioactive phenolic compounds amenable to further purification.

Subfractionation of CF3 using CCC techniques yielded 229 fractions, which were consolidated into five combined subfractions (CSF1–CSF5) based on TLC analysis. bioactivity screening revealed that CSF1 exhibited the highest DPPH and substantial alpha-glucosidase inhibition, demonstrating a favorable bioactivity profile for phytochemical characterization. CSF2 and CSF3 showed moderate DPPH activities but relatively weak AGI, suggesting enrichment of antioxidant-active compounds with limited antidiabetic potential. CSF4 displayed minimal bioactivity in both assays, while CSF5 demonstrated the highest AGI activity but showed the lowest antioxidant activity.

### 2.3. LC-HRMS/MS Phytochemical Profiling of Selected Subfraction

**Figure 1:**
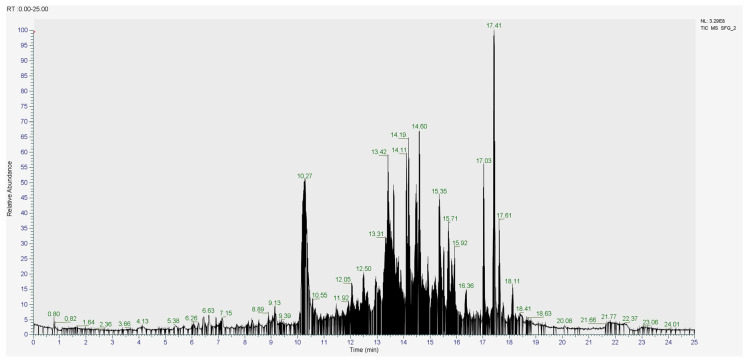
Chromatogram of CSF1 with T: 40 °C; Flow rate: 0.3 mL/min; Sample: 1 mg in 1 mL (MeOH 100%); Injection Volume: 3 µL.

The LC-HRMS/MS analysis of CSF1 from the ethanol extract of *S. polycephalum* leaf revealed a diverse array of bioactive secondary metabolites (Figure 1), comprising 12 tentatively identified compounds spanning multiple chemical classes. Among these, four compounds exhibited similarity scores greater than 90%, namely 4-methoxycinnamic acid, chrysin, formononetin, and caffeine (Table 4).

### 2.4. Molecular Docking Analysis

Both receptors (3L4Y and 6TYM) demonstrated excellent structural quality and docking protocol validity, with Ramachandran plots showing more than 87% of residues in the most favored region, ERRAT values ranging from 94.7% to 99.6%, and the majority of residues achieving Verify 3D scores of ≥0.2. Protocol validation yielded RMSD values of 1.28 Å for 3L4Y and 0.58 Å for 6TYM, both of which are substantially below the 2.0 Å threshold, confirming the high reproducibility and stability of the predicted binding poses. As shown in Table 5, phenolic and flavonoid constituents showed stronger experimental support, including higher similarity scores in LC-HRMS/MS identification and superior antioxidant and alpha-glucosidase inhibitory activities.

Drug-likeness was analyzed according to Lipinski’s Rule of Five, as summarized in Table 6, and revealed substantially different pharmacokinetic profiles across compounds. Chrysin and 4-methoxycinnamic acid demonstrated full compliance, positioning them as favorable candidates for oral development. Formononetin exhibited a single Log P violation (ΔLog P = +6.18), reflecting the trade-off between enhanced binding and lipophilicity, necessitating solubility-enhancement formulation strategies. Chrysin emerged as the optimal lead, combining Lipinski compliance with dual-target efficacy.

In silico ADMET assessment (Table 7) indicated favorable profiles for key compounds, particularly phenolic flavonoids. Chrysin exhibited the most balanced pharmacokinetic profile with high bioavailability, moderate distribution, acceptable protein binding, and absence of CYP3A4/CYP2D6 inhibition, suggesting minimal drug–drug interaction risk. No hepatotoxicity or mutagenicity signals were predicted. Formononetin were identified as CYP3A4 substrates, indicating active hepatic metabolism without major toxicity alerts.

**Figure 2:**
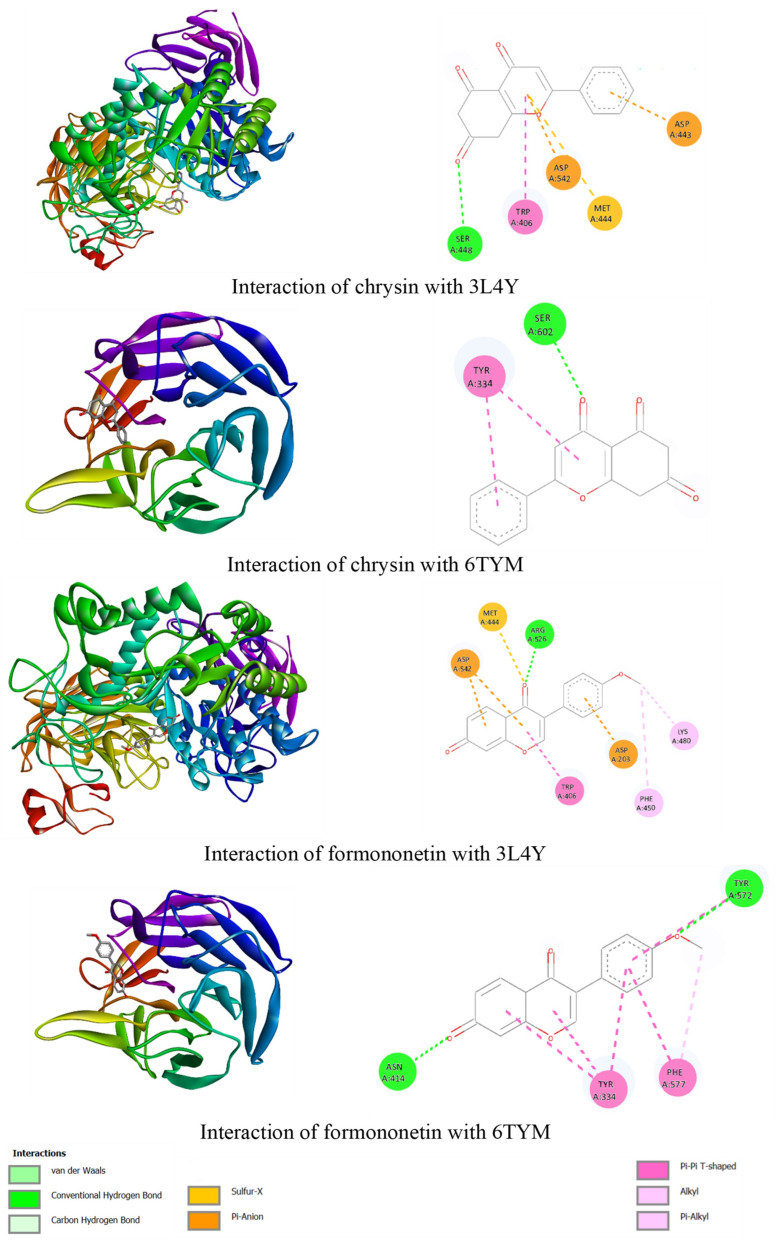
3D and 2D diagrams presenting the interaction of chrysin and formononetin as potential inhibitors with 3L4Y and 6TYM.

The binding of chrysin and formononetin to 3L4Y and 6TYM is illustrated in Figure 2. Chrysin binds alpha-glucosidase via SER A:448 hydrogen bonds and hydrophobic interactions, utilizing different sub-pockets than acarbose to demonstrate active site plasticity. For Keap1, chrysin binds SER A:602, competitively occupying the Nrf2-binding pocket and preventing its degradation, which explains chrysin’s documented antioxidant activity. Formononetin exhibited comparable binding affinity but compromised drug-likeness due to increased lipophilicity, making chrysin the more developmentally tractable lead candidate. 4-Methoxycinnamic acid demonstrated moderate binding with an exceptional pharmacokinetic profile. 

### 2.5. Molecular Dinamyc Analysis

**Figure 3:**
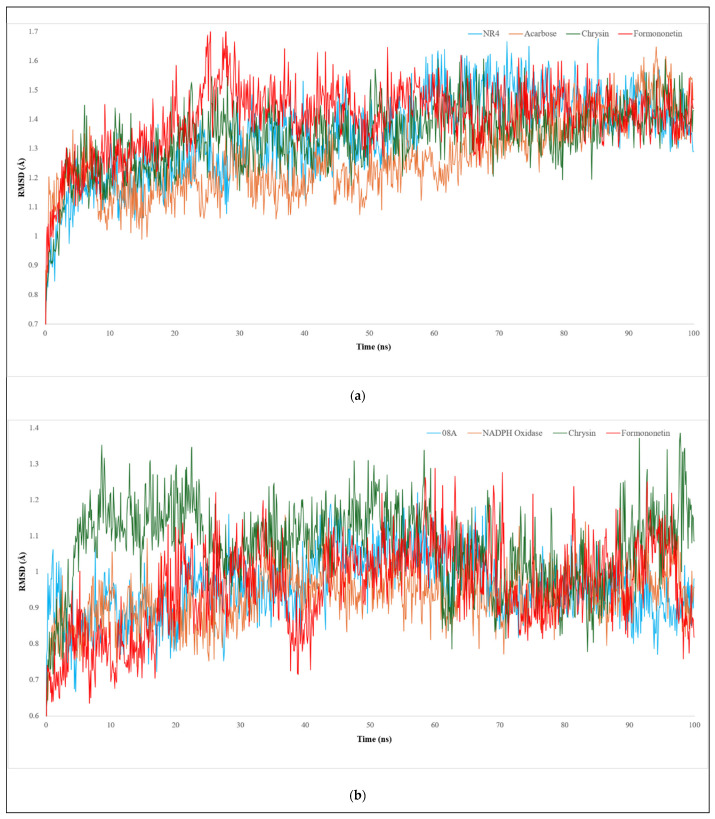
Graph of the global RMSD values of the simulation: (**a**) 3L4Y; (**b**) 6TYM.

Molecular dynamics analysis revealed that complex stability reflects the suitability of ligand-binding mechanisms for each target. Based on trajectory data (Figure 3) and (Table 8), the 3L4Y complex exhibited RMSD values of 1.264–1.401 Å, with chrysin (1.338 Å) comparable to the native ligand NR4 (1.345 Å), indicating preservation of binding orientation without significant conformational distortion, consistent with competitive inhibition in GH31 enzymes.

**Figure 4:**
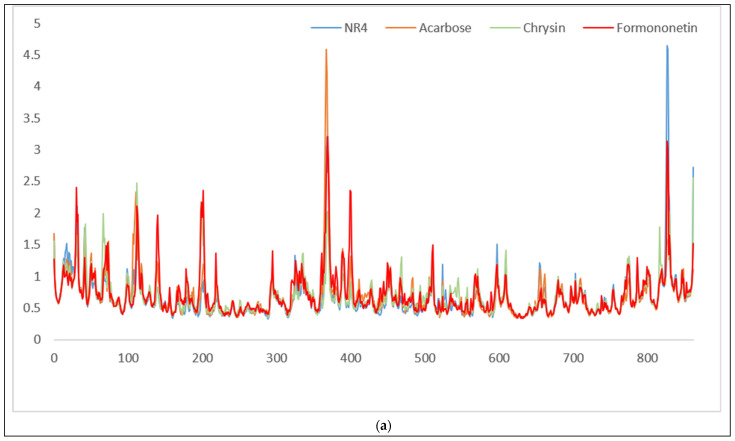

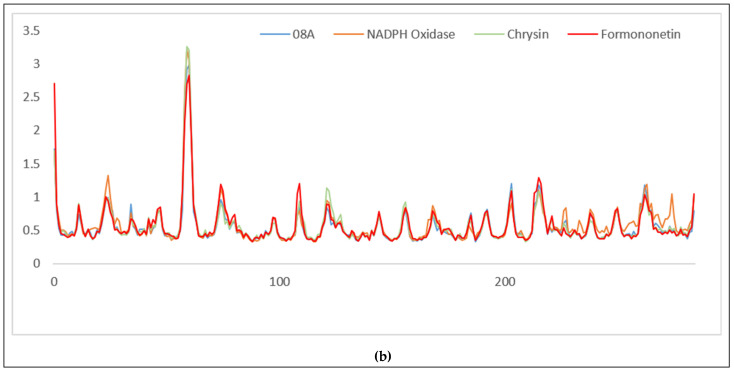
Graph of the global RMSF values of the simulation: (**a**) 3L4Y; (**b**) 6TYM.

In contrast, KEAP1 (6TYM) showed lower RMSD values (<1.0 Å), with formononetin (0.951 Å) closely matching the control ligand (0.955 Å), reflecting the structural rigidity of the Kelch domain. This trend was further supported by RMSF analysis (Figure 4; Table 9), where chrysin reduced residue fluctuations in ntMGAM relative to acarbose, while formononetin stabilized key residues in KEAP1, indicating ligand-induced restriction of local dynamics within the binding pocket.

MM-GBSA results (Table 10) demonstrated that all interactions were thermodynamically favorable (ΔG < 0). In ntMGAM, chrysin showed higher binding affinity than formononetin, mainly driven by van der Waals interactions, suggesting compatibility with the hydrophobic environment of the enzyme. In KEAP1, chrysin exhibited markedly stronger binding compared to formononetin, predominantly due to electrostatic contributions, which may reflect favorable interactions between the hydroxyl groups of chrysin and the protein environment.

## 3. Discussion

Table 1 showed that the leaf displayed the highest yield, This is consistent with the physiological role of the leaf as a photosynthetic center, where the shikimate pathway actively synthesizes phenolic compounds. Furthermore, the leaf’s constant exposure to environmental stressors, such as UV radiation and herbivores promotes the accumulation of defensive secondary metabolites [14]. Among the antioxidant assays employed, FRAP yielded the most pronounced response, particularly for leaf, attributable to its single electron transfer (SET) mechanism operating optimally at acidic pH (3.6), favoring hydroxyl-rich phenolics [18]. In contrast, the hydrogen atom transfer (HAT)-based DPPH assay is more selective for low-molecular-weight antioxidants and is sensitive to pH and solvent polarity [19]. The CUPRAC assay, also a SET-based mechanism but performed near neutral pH, complements these results by detecting a broader structural diversity of phenolics [20]. The combined use of these assays provides a more comprehensive antioxidant profile. In terms of AGI activity, the leaf extract demonstrated significant antioxidant activities, as indicated by the AEAC values across all three assays (DPPH, FRAP, and CUPRAC). This antioxidant activity was further supported by its high AGI activity, which reached 52,145.16 ± 801.54 mg AEAGIC/g extract. This value greatly surpasses typical activities reported for other botanical α-glucosidase inhibitors, highlighting the pharmacological importance of *S. polycephalum* leaf. The high AGI activity of the leaf extract can be attributed to its elevated phenolic content, as phenolic compounds are known to inhibit α-glucosidase by forming hydrogen bonds and engaging in hydrophobic interactions within the enzyme’s catalytic site, thereby reducing its enzymatic activity [21,22]. Based on these comprehensive bioactivity profiles, leaf extracts were selected for further bioassay-guided fractionation studies due to their superior phytochemical content and dual antioxidant–antidiabetic potential.

Based on Table 2, the ethanol extract exhibited the highest TPC, while the ethyl acetate extract showed the highest TFC, indicating selective extraction of moderately polar flavonoid aglycones. In contrast, the lower flavonoid content in the ethanol extract suggests that ethanol predominantly extracts polar phenolics and glycosylated flavonoids, while ethyl acetate is more effective at extracting fewer polar aglycones. These findings highlight the differences in solvent selectivity, which play a crucial role in the phytochemical profile of plant extracts and their potential bioactivities [23,24]. Furthermore, at the fraction level, the highest TPC and TFC were found in CF3. This fraction likely contains a concentrated mixture of phenolic acids and flavonoid aglycones that remain after the fractionation process. Fractionation techniques are widely used to isolate and concentrate specific bioactive compounds such as phenolic acids and flavonoids, which are often more concentrated in specific fractions following solvent extraction and separation processes [25].

As shown in Table 3, the superior bioactivity of the ethanol extract is probably due to the synergistic contribution of multiple polar phenolic compounds, including phenolic acids, flavonoid glycosides, and tannins, which are efficiently extracted by ethanol [16,26]. This suggests that the polarity of the solvent plays a significant role in enhancing the bioactive potential of the extract by selectively extracting bioactive compounds. At the subfraction stage (CSF), the pattern observed indicates that alpha-glucosidase inhibition (AGI) activity does not always correlate with antioxidant activity as measured by the DPPH assay. SFG5, the most polar subfraction, exhibited the highest AGI activity but showed weaker radical scavenging capacity in the DPPH assay. Conversely, SFG1 demonstrated the strongest antioxidant activity while still maintaining significant AGI potential. These differences suggest that the structural determinants controlling both biological activities are not entirely identical. The high antioxidant and AGI activities observed simultaneously in SFG1 imply the presence of phenolic constituents with dual bioactivity. Comparatively, semi-polar fractions like SFG1 tend to exhibit superior antioxidant performance compared to more polar fractions, suggesting that the main bioactive components in *S. polycephalum* leaf extract are concentrated within a medium polarity range. Chemically, semi-polar polyphenols, particularly flavonoid aglycones or partially methylated flavonoids, possess a favorable combination of high hydrogen-donating capacity due to abundant hydroxyl groups and adequate lipophilicity, facilitating effective interactions with enzyme active sites. Structure–activity relationship studies have shown that flavonoids with conjugated π-systems and optimal hydroxyl substitutions tend to exhibit strong antioxidant and AGI potential [27,28].

Phytochemical investigations of various *Syzygium* species have consistently identified flavonols such as quercetin, kaempferol, and myricetin, along with their glycosides and derivatives, as major constituents in leaf and related extracts. These compounds have been detected in semi-polar extracts/fractions of *S. malaccense* and *S. cumini* using chromatographic and mass spectrometric techniques, and are widely recognized for their strong hydrogen donating capacity and contribution to antioxidant and enzyme inhibitory activities. The presence of such flavonols in semi-polar fractions supports their significant contribution to the biological activities observed in *Syzygium* leaf extracts [29,30,31].

Table 4 showed four compounds with similarity scores greater than 90%. Notably, the three phenolic compounds—4-methoxycinnamic acid, chrysin, and formononetin—are generally present in relatively low abundance within most *Syzygium* species. Phytochemical investigations across representative species such as *S. cumini*, *S. aromaticum*, and *S. polyanthum* consistently demonstrate that the genus is predominantly characterized by major classes of secondary metabolites, such as quercetin, myricetin, tannins, and essential oil constituents (e.g., eugenol and related terpenoids) [30]. The relatively higher detection of these minor phenolics in *S. polycephalum* suggests a distinct phytochemical signature that may contribute to its observed bioactivity.

4-Methoxycinnamic acid emerged as the most abundant compound with minimal mass error 0.03 ppm. This phenylpropanoid derivative belongs to the hydroxycinnamic acid family, which exhibits well-documented antioxidant and antidiabetic properties. The mechanism of action involves stimulation of insulin secretion through KATP-independent pathways and cAMP-dependent modulation of calcium influx in pancreatic β-cells [32]. In animal models, *p*-methoxy cinnamic acid (structurally related to 4-methoxy cinnamic acid) has demonstrated glucose-lowering effects without inducing hypoglycemia, suggesting a safe therapeutic profile [33]. Its antioxidant properties stem from the resonance stabilization of phenoxy radicals following hydrogen abstraction, with the electron-donating methoxy substituent further stabilizing the radical intermediate [34].

4-Methoxycinnamic acid and related hydroxycinnamic acid derivatives are widespread in the plant kingdom but have limited documentation specifically within the *Syzygium* genus. Ellagic acid, gallic acid, chlorogenic acid, ferulic acid, and caffeic acid dominate the phenolic acid profiles of *S. aromaticum*, *S. jambos*, *S. samarangense*, *S. malaccense*, and *S. cumini*. The detection of 4-methoxycinnamic acid in *S. polycephalum* may indicate species-specific O-methylation capacity in the phenylpropanoid biosynthetic pathway, potentially serving as a distinctive chemotaxonomic feature [35,36,37].

Chrysin, a flavone with the molecular formula C_15_H_10_O_4_, was identified as the compound with the highest similarity. Chrysin is defined by the absence of B-ring hydroxyl groups, which influences its biological activity profile. Although reports on its potency relative to acarbose are inconsistent [38,39]. This study demonstrates that chrysin, identified in the *S. polycephalum* leaf extract, exhibits strong AGI activity. This variability may reflect differences in enzyme sources, assay conditions, and chrysin concentrations tested. The AGI action of chrysin involves reversible binding at the active site, hindering substrate access and subsequent glucose production [40]. Chrysin has been tentatively detected at low levels in complex herbal preparations and leaf infusions containing bay leaf (*S. polyanthum*), which are known to be rich in flavonoids and other phenolic constituents [41,42]. The presence of chrysin in *S. polycephalum,* as revealed by LC–HRMS/MS profiling, may therefore represent a species-specific chemotaxonomic marker or the outcome of unique biosynthetic or ecological adaptations, potentially influenced by environmental factors, endophytic microorganisms, or local metabolic regulation within this species [30,43].

Formononetin, an O-methylated isoflavone represents another important flavonoid constituent. Isoflavones are known for their estrogenic activity and diverse biological effects, including antioxidant and enzyme inhibitory activities [44,45,46]. Formononetin has been reported to exhibit moderate AGI, with its activity influenced by the methylation pattern on the B-ring [15,47]. Formononetin, within the genus *Syzygium* (Myrtaceae), represents a notable phytochemical anomaly that has been tentatively confirmed in specific species such as *S. samarangense* [48], *S. polyanthum* [49], and *S. aromaticum* [50] using sensitive modern analytical techniques, including LC-MS/MS. Although the genus is predominantly characterized by flavonols (quercetin, myricetin), phenolic acids, and terpenes (eugenol) [30,36], these studies have shown that formononetin is present as a minor constituent that potentially contributes synergistically to the antidiabetic and antioxidant activities of the extract [51,52].

Caffeine, a purine alkaloid typically associated with *Coffea* and *Camellia* species, was identified in *S. polycephalum* with a high similarity score. While its presence in the *Syzygium* genus is less commonly reported than in other Myrtaceae, recent phytochemical screenings have detected trace alkaloids and caffeine in specific fractions of *Syzygium* species, such as *Syzygium zeylanicum,* where it acts as a minor constituent alongside phenolic compounds [53]. Although caffeine lacks the phenolic hydroxyl groups typically associated with antioxidant activity, its capacity to scavenge free radicals is attributed to its electron-rich purine ring structure containing conjugated double bonds. Computational and kinetic studies suggest that the C8-N9 bond and the conjugated C=C/C=N system facilitate radical adduct formation (RAF), allowing effective delocalization of unpaired electrons and neutralization of reactive species, particularly hydroxyl radicals [54,55].

Based on Table 5, Table 6 and Table 7, chrysin and formononetin emerged as the most promising dual-acting agents, exhibiting consistently strong binding to both targets alongside favorable experimental and pharmacokinetic profiles. This multi-target potency positions chrysin and formononetin as attractive candidates for dual-activity drug development [56].

As illustrated in Figure 2, chrysin’s binding to both alpha-glucosidase and Keap1 demonstrates a unique dual inhibition mechanism. While chrysin has been extensively studied in isolation, this is the first report showing its dual enzyme inhibition in the specific phytochemical context of *S. polycephalum*. The binding of chrysin to SER A:448 and SER A:602 positions it as a promising candidate for dual-target drug development, particularly for diseases related to both oxidative stress and diabetes. On the other hand, formononetin, although it exhibits comparable binding affinity, has compromised drug-likeness due to increased lipophilicity, which makes chrysin the more favorable candidate for further development. 4-Methoxycinnamic acid demonstrated moderate binding with an exceptional pharmacokinetic profile, positioning it as an attractive candidate for bioavailability-driven optimization. These findings collectively demonstrate that lead prioritization requires balancing binding affinity, drug-likeness, and pharmacokinetic properties (Table 6 and Table 7).

As shown in Figure 3 and Table 8, the RMSD values for 3L4Y suggest that chrysin maintains a binding orientation similar to the native ligand NR4, indicating stable complex formation and competitive inhibition of GH31 enzymes. The relatively higher RMSD values for 3L4Y (1.338 Å for chrysin) compared to KEAP1 (0.951 Å for formononetin) also indicate that the latter complex exhibits more structural rigidity, as seen in Figure 4 and Table 9, which show that formononetin’s binding to KEAP1 stabilizes the Kelch domain effectively. These observations align with previous findings on the structural integrity of enzyme-ligand interactions, particularly for enzymes involved in oxidative stress and metabolic regulation [53,57].

The MM-GBSA results, summarized in Table 10, further confirm that chrysin exhibits superior binding affinity across both targets, as evidenced by its stronger binding energy (−9.144 kcal/mol for ntMGAM and −13.534 kcal/mol for EAP1). This is largely due to van der Waals and electrostatic interactions, respectively, suggesting that chrysin is better suited to interact with the hydrophobic and polar environments of both target sites. In comparison, formononetin, despite showing moderate binding affinity, presents a less favorable pharmacodynamic profile due to its lower affinity and compromised drug-likeness, as indicated by the weaker thermodynamic scores and binding interactions. These findings emphasize the potential of chrysin as a more promising candidate for drug development targeting both GH31 enzymes and KEAP1, in contrast to formononetin, which exhibits less favorable properties overall [57,58,59].

Despite the promising findings obtained from the integrated in vitro and in silico analyses, this study has several limitations. The metabolite identification was based on tentative LC–HRMS/MS annotation and database matching without isolation and full structural elucidation. Molecular docking, ADMET prediction, and molecular dynamics simulations only provide computational predictions, which require further experimental validation. Future studies should focus on compound isolation, mechanistic validation, and pharmacological evaluation to confirm the therapeutic potential of *S. polycephalum* metabolites.

## 4. Materials and Methods

### 4.1. General Instrumentation and Reagents

The instruments used in this study included the following components: analytical balance (Mettler Toledo^®^, Greifensee, Switzerland), a multiskan skyhigh microplate spectrophotometer (Thermo Scientific^®^, Waltham, MA, USA), 96-well microplates (Corning®, Corning, NY, USA), micropipettes (Finnpipette^®,^ Thermo Fisher Scientific, Waltham, MA, USA), UV lamp (Camag^®^, Muttenz, Switzerland), rotary evaporator (Büchi Rotavapor R-100, Flawil, Switzerland), TLC silica gel 60 F254 (Merck^®^, Darmstadt, Germany), UV-Visible spectrophotometer (Shimadzu^®^ UV-1800, Kyoto, Japan), liquid chromatography system (Thermo Scientific™ Vanquish™ UHPLC Binary Pump, Waltham, MA, USA) and Orbitrap high-resolution mass spectrometer (Thermo Scientific™ Q Exactive™ Hybrid Quadrupole-Orbitrap™, Waltham, MA, USA). For testing total phenol and flavonoid content, the main reagents were gallic acid (Sigma-Aldrich^®^, St. Louis, MO, USA), quercetin (Sigma-Aldrich^®^, St. Louis, MO, USA), Folin–Ciocalteu reagent (Sigma-Aldrich^®^, St. Louis, MO, USA), and AlCl_3_ (Merck^®^, Darmstadt, Germany). In assessing antioxidant activity, the primary reagents were 2,4,6-tris(2-pyridyl)-s-triazine (TPTZ) (Sigma-Aldrich^®^, St. Louis, MO, USA), 2,2-diphenyl-1-picrylhydrazyl (DPPH) (Sigma-Aldrich^®^, St. Louis, MO, USA), ascorbic acid (Sigma-Aldrich^®^, St. Louis, MO, USA), and neocuproine (Sigma-Aldrich^®^, St. Louis, MO, USA). For fractionation, the main material were silica gel 60 H 5–40 μm (Merck^®^, Darmstadt, Germany), silica gel 60 02–05 mm (Merck^®^, Germany), Silica gel 60 0.063–0.200 mm (Merck^®^, Darmstadt, Germany). For alpha-glucosidase inhibitory (AGI) activity, the key reagents were alpha-glucosidase enzyme (Sigma-Aldrich^®^, St. Louis, MO, USA) and p-nitrophenyl-α-D-glucopyranoside (pNPG) substrate (Sigma-Aldrich^®^, St. Louis, MO, USA).

### 4.2. Material

Plant materials of *S. polycephalum* (leaves, twigs, seeds, and fruits) were collected from West Java, Indonesia. The plant was identified and authenticated at the Jatinangor Herbarium, Plant Taxonomy Laboratory, Department of Biology, Faculty of Mathematics and Natural Sciences, Padjadjaran University, Bandung-Sumedang. A voucher specimen (No.43/HB/12/2023) was deposited for reference.

The collected plant parts were subjected to wet sorting to remove impurities and damaged material, followed by washing under running water. The cleaned materials were sliced and dried in a hot air oven at 40–45 °C until a constant weight was achieved. The dried plant material was stored in airtight containers under dry conditions for further processing.

### 4.3. Extraction, Fractionation and Subfractionation of Selected Plant Parts

Extraction was carried out on the leaf, twig, seed, and fruit of *S. polycephalum* using reflux with ethanol as the solvent. A rotary evaporator was used to concentrate the extracts until they became viscous. Subsequently, each extract was analyzed to determine total flavonoid (TFC) and total phenolic (TPC) contents, and subjected to in vitro biological activity assays, including antioxidant and AGI activities. Successive extraction of the selected plant parts was carried out using reflux with *n*-hexane, ethyl acetate, and ethanol. Fractionation was performed using vacuum liquid chromatography (VLC) with silica gel 60 H 5–40 μm as the stationary phase and gradient elution of n-hexane-chloroform-methanol combination (from 50:0:0 to 0:0:50) as the mobile phase. Subfractionation was performed using classic column chromatography (CCC) with silica gel 60 (0.063–0.200 mm) as the stationary phase and isocratic elution with chloroform-ethyl acetate-methanol (7:2:2) as the mobile phase.

### 4.4. Quantification of Total Phenol Content (TPC)

The Folin–Ciocalteu assay was applied to quantify total phenolic content, following the protocol of Pourmorad et al. [60]. The assay used gallic acid as the reference standard, prepared in various concentrations in methanol. Gallic acid standard aliquots (50 μL) were combined with 10% Folin–Ciocalteu reagent (500 μL) and 1 M sodium carbonate (400 μL), and the mixture was incubated for 30 min. Extract samples were dissolved in methanol and filtered through Whatman filter paper. Samples were then treated identically to the standards. The absorbance was evaluated using a UV-Vis spectrophotometer at 765 nm. TPC was calculated using the linear regression equation derived from the gallic acid calibration curve and reported as mg GAE/g extract.

### 4.5. Quantification of Total Flavonoid Content (TFC)

TFC was quantified by a colorimetric assay following the modified protocol of Chang et al. [61], with quercetin as the reference standard. Quercetin standard solutions (100 μL at various concentrations) were combined with pro-analysis methanol (300 μL), 10% AlCl_3_ (20 μL), 1 M sodium acetate (20 μL), and distilled water (560 μL). The mixture was then incubated for 30 min at room temperature. Absorbance was measured at 415 nm. Extract samples were prepared and treated identically to the standards. The linear regression equation of the quercetin calibration curve was employed to determine the TFC and expressed as mg quercetin equivalents per g of extract (mg QE/g extract) [62].

### 4.6. DPPH (2,2-Diphenyl-1-Picrylhydrazyl) Assay

Assessment of antioxidant activity was carried out using the DPPH method, as described by Celep et al. [63]. A 50 µg/mL DPPH solution and 200 µg/mL ascorbic acid standard were prepared in methanol, while the sample extracts were prepared at 10,000 µg/mL. Triplicate serial dilutions of the standard were mixed with DPPH solution (125 μL sample + 750 μL DPPH), incubated for 30 min at room temperature under dark conditions before measuring absorbance at 517 nm.

### 4.7. FRAP (Ferric Reducing Antioxidant Power) Assay

FRAP activity was assessed based on the procedure reported by Özyürek et al. [64], with minor modifications. The FRAP reagent was freshly prepared (FeCl_3_·6H_2_O, TPTZ, and acetate buffer in a 1:1:10 ratio). Ascorbic acid (20 mg/100 mL methanol) was used as the standard and diluted to various concentrations. Aliquots of 50 μL ascorbic acid were then reacted with 3 mL of FRAP reagent and 950 μL of distilled water. Absorbance was recorded at 595 nm following incubation for 30 min at room temperature.

### 4.8. CUPRAC (Cupric Ion Reducing Antioxidant Capacity)

The cupric ion reducing antioxidant capacity (CUPRAC) reagent (CuCl_2_–neocuproine, 1:1) was diluted in ammonium acetate buffer (pH 7.0) to 100 μg/mL. Ascorbic acid standards were mixed with 250 µL buffer and 750 µL CUPRAC reagent. Absorbance was measured at 450 nm.

The antioxidant activity of the extracts was determined using the ascorbic acid equivalent antioxidant capacity (AEAC) approach [64]. For the DPPH assay, the percentage inhibition of radicals was calculated for each sample and converted into mg AEAC per gram of extract using a calibration curve of ascorbic acid and the sample concentration. For the FRAP and CUPRAC assays, absorbance values were first converted to transmittance (%T), and the percent capacity was calculated as 100—%T to represent the increase in antioxidant potential. These percent capacity values were then converted into mg AEAC per gram of extract (mg AEAC/g) using the corresponding calibration curves and sample concentrations.

### 4.9. Alpha-Glucosidase Inhibitory (AGI) Activity Assay

The AGI activity was assessed using the protocol of Vonia et al. [65], with minor modifications. In a 96-well plate, 30 μL of sample, 36 μL of 0.1 M phosphate-buffered solution (PBS, pH 6.8), and 17 μL of 6 mM pNPG were combined and preincubated for 5 min at 37 °C. After adding 17 μL of alpha-glucosidase (0.2 U/mL), the mixture was incubated for 15 min at 37 °C to initiate the enzymatic reaction. Reaction termination was achieved by adding 100 μL of 200 mM Na_2_CO_3_ to each well. Absorbance values at 400 nm were obtained using a microplate reader multiskan skyhigh (Thermo Scientific®, Waltham, MA, USA). Acarbose served as the positive control, and the negative control contained no inhibitor. A calibration curve was generated using percentage inhibition values obtained from serial concentrations of acarbose. The analysis for acarbose was performed in six repetitions. The inhibitory activity of alpha-glucosidase was determined using the formula: % Inhibition = ((B1 − B2)/B1) × 100. B1 represents the absorbance of the blank minus the absorbance of the control blank, and B2 represents the absorbance of the sample minus the absorbance of the control sample. The blank consisted of PBS + pNPG + enzyme + Na_2_CO_3_, while the control blank contained PBS + Na_2_CO_3_ (without enzyme), and the control sample contained extract + PBS + Na_2_CO_3_ (without enzyme). The same procedure was applied to the sample extracts. The AGI activity of the samples was expressed as mg of acarbose equivalent alpha-glucosidase inhibitory capacity per g of sample (mg AEAGIC/g sample), calculated using the regression equation from the acarbose calibration curve.

### 4.10. LC-HRMS/MS Analysis of the Subfraction

Phytochemical analysis was performed using LC-HRMS/MS provided by Markherb, Bandung, Indonesia. Chromatographic separation was achieved on a phenyl-hexyl column (100 mm × 2.1 mm ID × 2.6 μm) maintained at 40 °C with an injection volume of 3 μL. The mobile phase consisted of MS-grade water containing 0.1% formic acid (A) and MS-grade methanol containing 0.1% formic acid (B). Gradient elution was performed at a 0.3 mL/min flow rate, starting from 5% B and increasing to 90% over 16 min, held at 90% B for 4 min, then returning to initial conditions at 25 min. Mass spectrometry detection employed positive electrospray ionization mode with the following parameters: capillary voltage of 3.30 kV, capillary temperature of 320 °C, and scan range of 66.7–1000 *m*/*z*. Sample aliquots (1 mg) were dissolved in pure methanol (1 mL) and filtered through a 0.22 μm organic membrane filter before injection. Compounds were identified using MzCloud (https://www.mzcloud.org/), ChemSpider (https://www.chemspider.com/), and PubChem databases (https://pubchem.ncbi.nlm.nih.gov/).

### 4.11. In Silico Study

The alpha-glucosidase (PDB ID: 3L4Y) and Keap1 (PDB ID: 6TYM) receptors were obtained from the Protein Data Bank and prepared using Discovery Studio 2021 Client. Test compounds were processed with MarvinSketch 23.13 and energy-minimized. Molecular docking was performed using AutoDockTools 1.5.7 with the Lamarckian Genetic Algorithm (100 runs), validated by re-docking natural ligands (RMSD ≤ 2 Å). Binding energies were analyzed as the primary affinity parameter. ADMET properties were predicted using the pkCSM server (https://biosig.lab.uq.edu.au/pkcsm/prediction, accessed on 30 October 2025), and drug-likeness was assessed via Lipinski’s Rule of Five.

Molecular dynamics (MD) simulations were performed for 100 ns using OpenMM 8.5.2 version with GPU acceleration to evaluate the structural stability of protein–ligand complexes under physiological conditions. The protein and ligand were parameterized using the FF19SB and GAFF2 force fields, respectively. The system was solvated in a TIP3P water model and neutralized with 0.15 M Na^+^ ions. Following energy minimization, the system was equilibrated at 310 K prior to production runs under NPT conditions to monitor conformational dynamics. Trajectory analyses were conducted using root mean square deviation (RMSD) and root mean square fluctuation (RMSF). Binding free energy was estimated using the MM-GBSA approach to assess interaction strength and complex stability, as described in recent studies [6,66].

### 4.12. Statistical Analysis

The results of each assay, including TPC, TFC, antioxidant activity, and AGI activity were presented as mean ± standard deviation. Statistical significance was determined using Tukey’s post hoc, Kruskal–Wallis, and Mann–Whitney tests, with *p* < 0.05 considered statistically significant.

## 5. Conclusions

The ethanol extract of *S. polycephalum* leaf exhibited superior antioxidant and α-glucosidase inhibitory activities, which can be attributed to its high phenolic and flavonoid contents. Notably, this non-edible plant part demonstrated greater bioactivity than the traditionally consumed fruit, highlighting its potential as a more effective source for pharmaceutical development. The extract also showed stronger potential as a raw material for herbal formulations compared to its fractions and subfractions. LC–HRMS/MS analysis identified 12 tentative bioactive compounds, including four metabolites: 4-methoxycinnamic acid, chrysin, formononetin, and caffeine. Molecular docking and in silico pharmacokinetic evaluation further supported chrysin and formononetin as promising dual-target inhibitors with favorable safety profiles and drug-like properties.

Molecular dynamics simulations confirmed stable protein–ligand interactions and favorable energetics. Chrysin showed superior stability and binding affinity across both targets, driven by van der Waals and electrostatic interactions, whereas formononetin was weaker. Together with the unique phytochemical profile and renewable availability of *S. polycephalum* leaf, these findings support their potential as sustainable dual-acting agents for type 2 diabetes.

## Figures and Tables

**Table 1:** Determination of TPC, TFC, antioxidant, and AGI activities of ethanolic extracts from different parts of *S. polycephalum*.

Plant Parts	TPC (mg GAE/g)	TFC (mg QE/g)	Antioxidant Activity (mg AEAC/g)			AGI Activity (mg AEAGIC/g)
			DPPH	FRAP	CUPRAC	
Leaves	457.89 ± 12.10 ^a^	11.08 ± 1.10 ^a^	683.21 ± 24.54 ^a^	1338.37 ± 7.04 ^a^	771.91 ± 8.78 ^a^	52,145.16 ± 801.54 ^a^
Twigs	270.93 ± 26.17 ^b^	9.46 ± 0.50 ^b^	345.17 ± 7.30 ^b^	524.05 ± 0.89 ^b^	649.53 ± 18.35 ^b^	20,661.57 ± 461.56 ^b^
Seeds	385.57 ± 64.14 ^c^	6.46 ± 0.71 ^c^	435.19 ± 4.95 ^c^	1220.40 ± 43.16 ^c^	630.03 ± 25.15 ^b^	7970.92 ± 604.23 ^c^
Fruits	63.74 ± 3.77 ^d^	1.77 ± 0.28 ^d^	61.92 ± 1.28 ^d^	147.95 ± 1.48 ^d^	279.40 ± 13.72 ^c^	5857.64 ± 194.59 ^d^

Data are presented as mean ± SD for six replicates measurements. ^a–d^ Different superscript letters within the same column indicate significant differences (*p* < 0.05). GAE = gallic acid equivalent; QE = quercetin equivalent; AEAC = ascorbic acid equivalent antioxidant capacity; AEAGIC = acarbose equivalent α-glucosidase inhibitory capacity.

**Table 2:** Determination of TPC and TFC of *S. polycephalum* leaf extract and fractions.

Samples	TPC (mg GAE/g)	TFC (mg QE/g)
n-Hexane	24.54 ± 0.48 ^a^	40.23 ± 1.12 ^a^
Ethyl acetate	36.80 ± 0.60 ^b^	83.69 ± 1.11 ^b^
Ethanol	309.64 ± 2.66 ^c^	8.79 ± 0.31 ^c^
CF1	2.37 ± 0.02 ^d^	ND
CF2	3.28 ± 0.06 ^e^	2.49 ± 0.09 ^d^
CF3	69.72 ± 0.49 ^f^	6.09 ± 0.16 ^e^

Data are presented as mean ± SD for six replicates measurements. ^a,b,c,d,e,f^ Different superscript letters within the same column indicate significant differences. CF: Combined fraction. ND = Not detected.

**Table 3:** Antioxidant activities and alpha-glucosidase inhibitory of *S. polycephalum* leaf extracts, fractions, and sub-fractions.

Extract	DPPH (mg AEAC/g)	FRAP (mg AEAC/g)	CUPRAC (mg AEAC/g)	AGI Activity (mg AEAGIC/g)
n-Hexane	52.15 ± 1.41	22.61 ± 0.66	55.85 ± 1.77	696.28 ± 144.68
Ethyl acetate	284.21 ± 2.7	166.19 ± 1.33	154.36 ± 1.01	224.02 ± 43.91
Ethanol	5919.35 ± 37.54	2093.01 ± 56.08	1287.72 ± 7.82	10,867.73 ± 303.43
CF1	30.711 ± 0.324	ND	ND	ND
CF2	35.90 ± 0.84	ND	ND	25.81 ± 8.71
CF3	337.82 ± 3.99	252.31 ± 5.03	203.29 ± 6.54	2235.91 ± 25.57
CSF1	239.04 ± 3.64	ND	ND	277.56 ± 6.69
CSF2	211.05 ± 2.80	ND	ND	68.33 ± 7.00
CSF3	186.74 ± 1.48	ND	ND	10.43 ± 1.25
CSF4	23.04 ± 0.53	ND	ND	26.60 ± 0.63
CSF5	68.08 ± 1.62	ND	ND	595.44 ± 7.35

Data are presented as mean ± SD for six replicates measurements. AEAC = ascorbic acid equivalent antioxidant capacity; AEAGIC = acarbose equivalent α-glucosidase inhibitory capacity; CF = Combined fraction; CSF = Combined subfraction; ND = Not detected.

**Table 4:** LC-HRMS/MS results of 12 tentative compounds of CSF1.

Proposed Compound	Retention Time (min)	Molecular Weight	Molecular Formula	Conc. (%)	Similarity (%)	Metabolite Class
4-Methoxycinnamic acid	15.088	178.063	C_10_H_10_O_3_	3.284	94.8	Phenolic acid
Chrysin	10.004	254.058	C_15_H_10_O_4_	0.286	99.9	Flavone
Formononetin	8.517	268.073	C_16_H_12_O_4_	0.489	93.2	Isoflavone
Caffeine	4.128	194.080	C8H_10_N_4_O_2_	1.382	99.7	Alkaloid
Stigmasterol	16.492	412.3708	C_29_H_48_O	1.084	89.2	Phytosterol
8,10-Dihydroxy-3-methoxy-9-[(1E)-3-methyl-1-buten-1-yl]-6-(2-methyl-1-propen-1-yl)-6H,7H-chromeno [4,3-b]chromen-7-one	14.626	434.17348	C_26_H_26_O_6_	0.477	88.8	Biflavonoids
(24Z)-3-Acetoxy-15-hydroxy-23-oxolanosta-7,9(11),24-trien-26-oic acid	15.429	526.33	C_32_H_46_O_6_	6.388	80.7	Triterpenoids
(3β,24R,24′R)-fucosterol epoxide	16.803	428.366	C_29_H_48_O_2_	0.792	77.7	Phytosterol
8-(3,4-Dihydroxyphenyl)-5-hydroxy-7-methoxy-6H-[1,3]dioxolo [4,5-h]chromen-6-one	9.135	344.053	C_17_H_12_O_8_	2.784	76	Coumarins
(E,E)-α-Farnesene	12.605	204.18787	C_15_H_24_	0.196	68.7	Sesquiterpene
2-(2,4-Dihydroxyphenyl)-3-[(2Z)-3,7-dimethyl-2,6-octadien-1-yl]-5,7-dihydroxy-6-(3-methyl-2-buten-1-yl)-2,3-dihydro-4H-chromen-4-one	15.717	492.25176	C_30_H_36_O_6_	0.264	62.8	Prenylated flavonoids
(24E)-3-Acetoxy-15,22-dihydroxylanosta-7,9(11),24-trien-26-oic	15.677	528.34592	C_32_H_48_O_6_	0.476	60.8	Triterpenoids

**Table 5:** Binding energy of Ki, H-bond, and hydrophobic interaction.

Compound	Protein	ΔG_binding_ (kcal/mol)	Ki (µM)	H-Bond	Hydrophobic Interaction
NR4 (native ligand)	3L4Y	−2.18	25,290	ARG A:202, LYS A:480, ASP A:203	-
08A (native ligand)	6TYM	−8.19	1.00	ARG A:415, ASN A:414, TYR A:334, ARG A:380, ASN A:382	PHE A:577, TYR A:334, ARG A:380
Acarbose (control ligand)	3L4Y	−4.47	526.26	GLN A:603, ASP A:542, ASP A:203	-
NADPH Oxidase-inhibitor (control ligand)	6TYM	−5.68	68.39	TYR A:334	ALA A:556, ARG A:336, PHE A:577, TYR A:572
4-Methoxycinnamic acid	3L4Y	−3.16	4820	ARG A:202, THR A:205	MET A:444, TRP A:406
	6TYM	−4.64	397.03	ASN A:382, TYR A:334, SER A:602	TYR A:572, ALA A:556
Chrysin	3L4Y	−5.98	41.49	SER A: 448	TRP A:406, MET A:444
	6TYM	−5.31	128.97	SER A:602	TYR A:334
Formononetin	3L4Y	−6.03	38.14	ARG A:526	MET A:444, TRP A:406, PHE A:450, LYS A:480
	6TYM	−5.20	154.87	ASN A:414, TYR A:572	TYR A:334, PHE A:557
Caffeine	3L4Y	−4.72	345.30	ARG A:202, THR A:204, THR A:205, ASP A:203, ASP A:542	LYS A:480, LEU A:473, THR A:204, SER A:448, MET A:444, TYR A:214
	6TYM	−4.26	750.38	ASN A:414	ARG A:380, TYR A:334

**Table 6:** Lipinski’s rule of five.

Compound	MW (≤500)	HBD (≤5)	HBA (≤10)	Log P (≤5)	RM (40–130)
Acarbose	645	14	19	−8.5	137.74
NADPH Oxidase-Inhibitor	373	1	5	2.85	108.85
4-Methoxycinnamic Acid	177	0	3	0.46	47.03
Caffeine	194	0	6	−0.35	49.21
Chrysin	252	0	4	1.62	67.38
Formononetin	267	2	4	7.80	73.22

MW: Molecule Weight; HBD: Hydrogen Bond Donor; HBA; hydrogen Bond Acceptor; RM: Refractory Molar.

**Table 7:** Pharmacokinetic profile prediction results.

Parameter	Compound					
	(a)	(b)	(c)	(d)	(e)	(f)
Intestinal Absorption (Log mol/L)	0	90.862	94.977	99.272	93.761	96.112
Caco-2 Permeability (Log Kp)	−0.717	1.097	1.236	1.115	0.945	1.253
VDss (log L/kg)	−0.833	0.349	−1.182	−0.595	0.403	−0.121
Fraction Unbound (Fu)	0.569	0.257	0.305	0.651	0.136	0.096
CYP3A4 (Substrate)	No	Yes	No	No	No	Yes
CYP3A4 (Inhibitor)	No	No	No	No	No	No
CYP2D6 (Substrate)	No	Yes	No	No	No	No
CYP2D6 (Inhibitor)	No	No	No	No	No	No
Total Clearance (log mL/min/kg)	0.619	0.201	0.766	0.193	0.405	0.258
Renal OCT2 Substrate	No	Yes	No	No	No	No
Hepatotoxicity	No	Yes	No	Yes	No	No
Ames Toxicity	No	No	Yes	No	No	No

(a) Acarbose; (b) NADPH Oxidase-Inhibitor; (c) 4-Methoxycinnamic acid, (d) Caffeine; (e) Chrysin; (f) Formononetin.

**Table 8:** RMSD values from molecular dynamics simulations.

Protein	Compound		RMSD (Å)	
		Mean	Minimal	Maximum
3L4Y	Native ligand (NR4)	1.345	0.782	1.676
	Control ligand (Acarbose)	1.264	0.832	1.647
	Chrysin	1.338	0.729	1.607
	Formononetin	1.401	0.784	1.734
6TYM	Native ligand (08A)	0.955	0.667	1.223
	Control ligand (NADPH Oxidase)	0.930	0.643	1.157
	Chrysin	1.074	0.627	1.385
	Formononetin	0.951	0.635	1.287

**Table 9:** RMSF values from molecular dynamics simulations.

Protein	Compound		RMSF (Å)	
		Mean	Minimal	Maximum
3L4Y	Native ligand (NR4)	0.676	0.329	4.650
	Control ligand (Acarbose)	0.696	0.338	4.583
	Chrysin	0.689	0.345	2.555
	Formononetin	0.717	0.341	3.204
6TYM	Native ligand (08A)	0.554	0.334	2.979
	Control ligand (NADPH Oxidase)	0.590	0.329	3.214
	Chrysin	0.568	0.330	3.256
	Formononetin	0.570	0.331	2.828

**Table 10:** MM-GBSA energy components.

Protein	Ligand	Energy Components (kcal/mol)						
		EVDW	EEL	EGB	ESURF	∆G Gas	∆G Solv	∆Total
3L4Y	Native (NR4)	−14.483	−21.032	17.505	−1.229	−35.515	16.276	−19.239
	Control (Acarbose)	−17.438	−64.911	73.686	−4.043	−82.350	69.642	−12.707
	Chrysin	−14.696	−8.742	16.280	−1.986	−23.439	14.294	−9.144
	Formononetin	−14.338	−3.863	13.971	−2.295	−18.251	11.676	−6.575
6TYM	Native (08A)	−32.424	−19.768	34.000	−4.184	−52.193	29.815	−22.377
	Control (NADPH Oxidase)	−26.858	−11.991	21.616	−2.637	−38.849	18.979	−19.870
	Chrysin	−18.500	−14.570	22.147	−2.612	−33.070	19.535	−13.534
	Formononetin	−17.391	0.169	17.124	−1.978	−17.222	15.146	−2.076

## Data Availability

Derived data supporting the findings of this study are available from the corresponding author on request.

## Supplementary Materials

The following supporting information can be downloaded at https://www.mdpi.com/article/10.3390/molecules31122106/s1: Certificate S1: Plant identification results. References [67,68,69] are cited in Supplementary Materials.
