# Supplementary material for: Bioactivity-Guided Identification of Metabolites from Syzygium polycephalum with Antioxidant and α-Glucosidase Inhibitory Activities
Source: Molecules. 2026 Jun 15;31(12):2106. doi: 10.3390/molecules31122106 (PMC13305720; doi:10.3390/molecules31122106)
Supplement: Supplementary file 1 [file molecules-31-02106-s001.zip › molecules-4331679-supplementary.pdf]

HERBARIUM JATINANGOR
LABORATORIUM TAKSONOMI TUMBUHAN
JURUSAN BIOLOGI FMIPA UNPAD
Gedung D2-212, Jl. Raya Bandung Sumedang Km 21 Jatinangor
Telp. 089689992695, email: phanerogamae@yahoo.com

LEMBAR IDENTIFIKASI TUMBUHAN
No.43/HB/12/2023

Herbarium Jatinangor, Laboratorium Taksonomi Tumbuhan, Jurusan Biologi FMIPA UNPAD, dengan ini menerangkan bahwa:

Nama : Ira Rahmiyani
NPM/NIDN : 30723003
Instansi : Sekolah Farmasi ITB.
Lokasi : Tasikmalaya.

Telah melakukan identifikasi tumbuhan, dengan No. Koleksi:
Tanggal Koleksi : 08 Desember 2023.
Lokasi : Tasikmalaya.

Hasil Identifikasi,
Nama Ilmiah : ***Syzygium polycephalum*** **(Miq.) Merr. & L.M.Perry**
Sinonim : *Eugenia polycephala* Miq.
Nama Lokal : Tanaman Kupa
Suku/Famili : Myrtaceae

Klasifikasi (Hirarki Taksonomi)

Kingdom	Plantae
Divisi	Magnoliophyta
Class	Magnoliopsida
Ordo	Myrtales
Famili	Myrtaceae
Genus	*Syzygium*
Species	*Syzygium polycephalum* (Miq.) Merr. & L.M.Perry

Referensi:
Cronquist, Arthur. 1981. *AnIntegrated System of Classification of Flowering Plants*. Columbia University Press. New York
The Plant List.*Website DuniaTumbuhan*.http://www.theplantlist.org/tpl1.1/record/kew-158489.
Backer, C. A. and Bakkuizen v/d Brink R. C Jr. 1963. *Flora of Java*. Wolter-Noordhoff NV. Groningen.

Jatinangor, 09 Desember 2023.

Identifikator,

LABORATORIUM TAKSONOMI TUMBUHAN
JURUSAN BIOLOGI FMIPA-UNPAD

Drs. Joko Kusmoro, M.P,
NIP. 19600801 199101 1 001
